# The Swedish theoretical framework of acceptability questionnaire: translation, cultural adaptation, and descriptive pilot evaluation

**DOI:** 10.1186/s12913-025-12855-x

**Published:** 2025-05-13

**Authors:** Maria Samuelsson, Marie-Louise Möllerberg, Merita Neziraj

**Affiliations:** Faculty of Health and Society, Department of Care Science, Malmö University, Jan Waldenströms gata 25, Malmö, 205 06 Sweden

**Keywords:** Acceptability, Complex healthcare interventions, Medical research council, Psychometric, Theoretical framework of acceptability, Translation

## Abstract

**Background:**

Successful complex healthcare interventions require evaluations of acceptability. Acceptability is suggested to impact intervention implementation, uptake, adherence, intended outcomes, and overall effectiveness. Namely, interventions that are not acceptable to those delivering or receiving them may hinder the key components from being delivered as intended or the recipients from engaging with the interventions as required. However, no validated questionnaire that evaluates acceptability was found in Swedish.

**Methods:**

We translated the generic Theoretical Framework of Acceptability questionnaire into Swedish, culturally adapted it, and conducted a descriptive pilot evaluation of its psychometric properties. The process involved iterative translation and cultural adaptation following the COSMIN checklist. The questionnaire underwent a forward–backwards translation and an evaluation of face and content validity by an expert panel of researchers. Thereafter, the face validity and comprehensibility of the translated version were evaluated using cognitive interviews and the think-aloud technique; this process was carried out in two rounds of interviews, each with a lay panel of healthcare professionals comprising intervention deliverers and receivers. Lastly, the Swedish version was piloted on 16 Swedish healthcare professionals who had received an educational intervention.

**Results:**

The evaluations of face validity, comprehensibility, and the descriptive pilot evaluation indicate a successful translation, cultural adoption, and usability of the Theoretical Framework of Acceptability questionnaire. The evaluation of content validity showed some problems with the validity of the scale and 7 out of 10 items was below threshold values.

**Conclusions:**

Overall, the Swedish Theoretical Framework of Acceptability questionnaire seems like a useful brief screening tool for the acceptability of healthcare interventions. The translation process revealed unresolved issues with content validity, possibly explained by the previously reported lack of consensus on the meaning of ‘acceptability’. Complementing free text answers or interviews could strengthen the understanding of any unclear questionnaire elements. Our findings support the generic Theoretical Framework of Acceptability questionnaire developers’ recommendations of continued cognitive interviewing and psychometric evaluations in any new setting. In addition, we recommend cross-measure validation between the existing acceptability questionnaires to help further refining the measurement of acceptability.

## Background

The Medical Research Council’s Framework for Developing and Evaluating Complex Healthcare Interventions emphasizes the role of evaluating *acceptability* in developing, evaluating, and implementing complex healthcare interventions (hereafter referred to as ‘interventions’) [1]. Medical Research Council suggest that acceptability has an impact on intervention implementation, uptake, adherence, intended outcomes, and overall effectiveness. For instance, interventions that are not acceptable to those delivering or receiving them may hinder key components from being delivered as intended or deter recipients from engaging with the interventions as required. Nevertheless, a systematic review has found an inconsistency across studies in evaluating and reporting acceptability and a lack of standardized and validated measurements [2]. Likewise, the Medical Research Council has reported a lack of definition of the term acceptability and a corresponding lack of consensus among researchers on its meaning and appropriate measurements [1]. Lack of consensus and validated measurements may negatively affect quality, introduce bias, and hinder the comparisons and synthesis of findings across interventions [2–4]. In response, Sekhon et al. developed the Theoretical Framework of Acceptability (TFA) which has been adopted rapidly in healthcare research (e.g. 1,658 citations in November 2024).

To enable measurements of acceptability, researchers have drawn upon the TFA framework and developed different questionnaires. For instance, Haydon et al. [5] developed the Digital Health Acceptability Questionnaire, and Sekhon et al. [5] developed a generic acceptability questionnaire (The TFA questionnaire). The latter has been translated to Spanish, showing satisfactory reliability [6]. In the current study, we focus on the generic TFA questionnaire developed by Sekhon et al. [5].

The TFA Questionnaire was developed based on a systematic review and feedback from key stakeholders and think-aloud interviews. The systematic review on which the questionnaire is based defines acceptability as ‘a multi-faceted construct that reflects the extent to which people delivering or receiving a healthcare intervention consider it appropriate, based on anticipated or experienced cognitive and emotional responses to the intervention’ [2]. Further, according to the TFA framework, acceptability consists of seven constructs: affective attitude, burden, ethicality, perceived effectiveness, intervention coherence, self-efficacy, and opportunity costs. Measuring the seven constructs through a questionnaire enables intervention developers to identify the aspects of interventions that require improvement [7]. In the questionnaire, each construct is represented by one item, apart from the constructs of affective attitude and ethicality, which are represented by two items from which the researchers choose the item that best fits the intervention. In addition to the seven constructs, the questionnaire also comprises an item assessing the general acceptability of the intervention. Thus, the questionnaire encompasses ten potential items. Each item is rated on a five-point numerical rating scale. The questionnaire is administered individually, and the responses are aggregated to yield a total score ranging from 8 to 40. Higher scores reflect greater levels of perceived acceptability.

The TFA questionnaire is designed to be usable across interventions and settings and across three temporal perspectives: before, during, and after participation in an intervention. Furthermore, assessments can be made by both intervention deliverers and receivers and across the research process of interventions: development, feasibility, piloting, and evaluation. The TFA questionnaire [7] has been used in several interventions and contexts. For instance, it has been used to evaluate follow-up regimens in cancer care [8], pharmacist-led IT-based interventions [9], new models of care in antenatal care [10] and a health coaching intervention for the prevention and management of type 2 diabetes [11]. However, we found no reports of a Swedish version of the TFA questionnaire. Therefore, we translated it from English to Swedish, culturally adapted it, evaluated the translated version’s face and content validity and comprehensibility, and piloted the questionnaire on a sample of 16 Swedish healthcare professionals. Prior to translation, permission was sought and granted from the authors of the original TFA questionnaire (Dr Sekhon and Professor Francis).

## Methods

This study aimed to translate and culturally adapt the Theoretical Framework of Acceptability questionnaire from English to Swedish and to pilot evaluate its descriptive psychometric properties. The research team who conducted the study (i.e. we, the authors) are registered nurses and researchers with experience in developing and evaluating complex healthcare interventions and conducting psychometric evaluations.

**Fig. 1:**
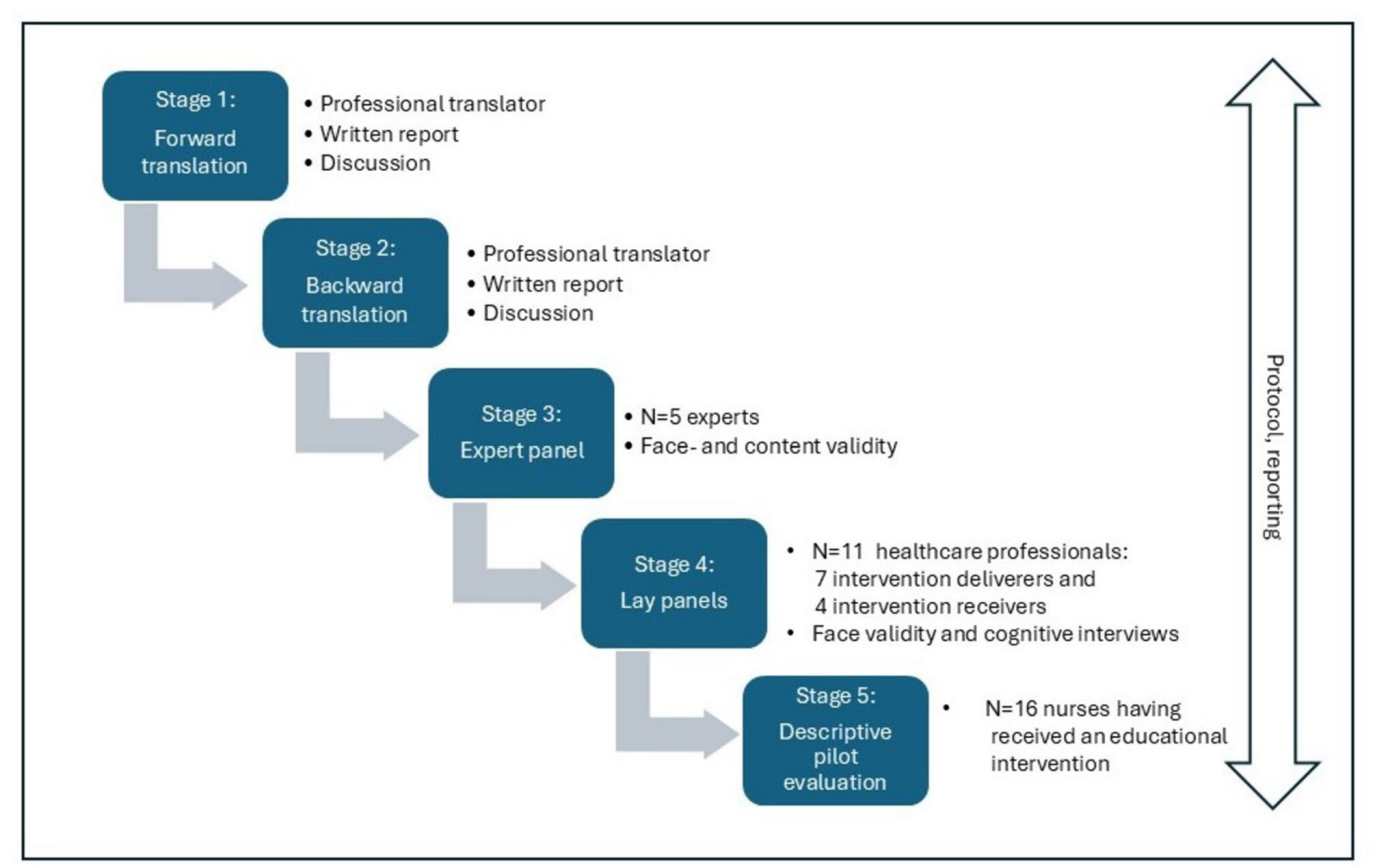
The process of translation and cultural adaptation, and descriptive pilot evaluation used in this study. Translation followed the COSMIN checklist and was influenced by the Beaton et al. [13] guidelines

The translation process was conducted in five stages, as illustrated in Fig. 1, following the COSMIN checklist [12] and influenced by the guidelines of Beaton et al. [11]. First, a forward–backwards translation was conducted iteratively, and the face and content validity of the translated version were evaluated by an expert panel of researchers. Thereafter, its face validity and comprehensibility were evaluated among two lay panels, comprising intervention deliverers and receivers, using cognitive interviews. Lastly, the translated questionnaire was piloted by a sample of 16 healthcare professionals who had received an educational intervention.

### Data collection

#### Participants

The inclusion criteria for the expert panel were being a researcher and having recognized expertise in developing, evaluating, or implementing complex healthcare interventions. The number of participants was based on Lynn’s recommendation of 3–10 participants [14]. For the lay panels, the inclusion criteria were being a healthcare professional in regional or municipal healthcare in southern Sweden. In addition to Lynn’s recommendation, the number of participants for the lay panels was also guided by Willis’s suggestion to recruit participants until no new uncertainties emerge during interviews [15]. The inclusion criteria for participating in the pilot evaluation of the Swedish TFA (S-TFA) questionnaire were being a healthcare professional employed at the involved clinics and having experience with the intervention used for evaluation. The sample size was based on recommendations by Beaton et al. [13] for piloting translated questionnaires using descriptive statistics.

#### Recruitment

Participants in the expert and lay panels were recruited using snowball sampling. Verbal and written information was provided to presumptive participants, and those interested were contacted by the research team and provided further information. Recruitment proceeded until the estimated sample size was reached (expert panel) and no further uncertainties were discovered during the interviews (lay panels). Participants in the pilot evaluation of the psychometric properties were recruited via a one-day paediatric nursing education for healthcare professionals held in the clinic where they were employed. After permission from the head of the department, all healthcare professionals who had participated in this education received an information letter with a link for further information on the study sent to their work e-mail addresses. After reading the study information, those interested could choose to proceed to a digital version of the S-TFA questionnaire. Three reminders were sent out. A total of 16 healthcare professionals responded to the questionnaires, resulting in a response rate of 31%.

#### Stage 1 and 2: forward–backward translation

The first two stages of the study involved forward–backward translation of the original TFA questionnaire. First, the questionnaire was translated from English to Swedish by one authorized translator, who documented any perceived difficulties or conceptual uncertainties. Thereafter, we discussed the translation and the translator’s written report until we reached consensus on a first draft. In the second stage, another authorised translator conducted a back translation of the first draft and made a written report. Lastly, we reviewed and discussed all versions and written reports until consensus was achieved on a second draft.

#### Stage 3: expert panel of researchers evaluating face and content validity

In the third stage, the expert panel individually evaluated the second draft’s face and content validity. Face validity was assessed through free-text replies. To evaluate content validity, the experts were asked to assess each item for relevance on a scale ranging from 1 = *not relevant* to 4 = *highly relevant*, as recommended by Polit and Beck [16].

#### Stage 4: lay panel of healthcare professionals evaluating face validity and comprehensibility

In the fourth stage, face validity was evaluated verbally by a lay panel of healthcare professionals: seven intervention deliverers and four intervention receivers. This process was conducted over two rounds; the first round involved a panel of six professionals, and the second one had a panel of five. Comprehensibility was evaluated verbally through cognitive interviews and the think-aloud method according to Willis [15]. The interviews were conducted by the research team, as we all have experience in carrying out qualitative and cognitive interviews. The panel in each round evaluated comprehensibility to gain insights into how the questionnaire respondents engage with the items through four cognitive processes—understanding the item, retrieving needed information, deciding how to respond, and providing their answers [15] —and to identify semantic and conceptual issues. In the first round, the participants were asked to recall an intervention they had delivered or received and the activity of the intervention (e.g. engage with) before the interview. The following are examples of interventions mentioned by the participants: the initiation of a screening programme, the change of a discharge plan, a new method of preparing children for a painful procedure, and an educational intervention. In the second round, the comprehensibility of different temporal phases (pre-, during, and post-delivering or receiving an intervention) was evaluated. To do so, we selected the intervention to assess. In both rounds, the participants were asked to read each item and response category aloud and explain how they understood it in relation to the intervention, as recommended in cognitive interviewing [15]. Further, they were asked to exemplify a response. Uncertainties were documented in a protocol based on French et al. [17] and Sekhon et al. [2]. Table 1 shows the protocol used to document the cognitive interviews. In between interviews, we discussed the findings. When all temporal phases were covered and the interviews revealed no uncertainties, following recommendations [15], the interview process ended.

**Table 1:**
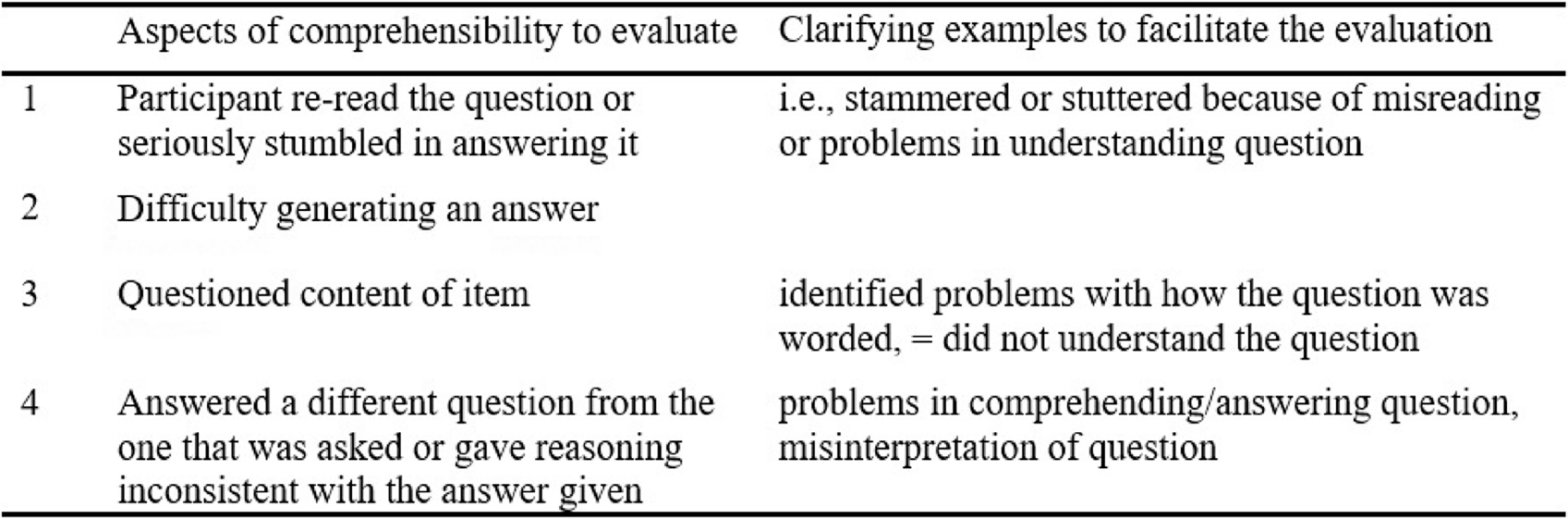
Protocol used to document the evaluation of comprehensibility

#### Stage 5: descriptive pilot evaluation of the Swedish TFA questionnaire on a sample of 16 Swedish intervention receivers

The psychometric properties of the S-TFA questionnaire were pilot evaluated in a sample of 16 Swedish healthcare professionals (nurses, nurse aids, and nutritionists) who had received an educational intervention in general paediatric care. The education was provided once a month, a full day at the time, over a nine-month period to all newly employed healthcare professionals at the clinic enrolled in this study. The aim of the intervention was to increase the participants knowledge in paediatric care. After the intervention, the participants answered the S-TFA questionnaire using a digital survey tool provided by the university where the research team is employed.

### Data analysis

To evaluate the S-TFA questionnaire, we analysed its face and content validity, comprehensibility, and conducted a descriptive pilot evaluation of data quality, targeting, and homogeneity.

#### Face and content validity

The face validity analysis was performed on the free-text replies of the expert panel and the verbal assessments of lay panels, following Streiner and Norman’s guide [18]. The content validity was based on the assessments of content relevance made by the expert panel using Item-Content Validity (I-CVI), Average Scale Content Validity (S-CVI/Ave), and Universal Agreement Scale Content Validity (S-CVI/UA) in accordance with Polit and Beck [16]. The recommended threshold of I-CVI is 1.00 and of S-CVI Ave is ≥ 0.90 [16].

#### Comprehensibility

Comprehensibility was analysed based on the cognitive interviews with the two lay panels. We discussed the interview protocols regularly, as recommended by Willis [15], in relation to Swedish healthcare and the conceptual ideas underpinning the original TFA questionnaire [2].

#### Descriptive pilot evaluation

The descriptive pilot evaluated data quality, targeting, and homogeneity of the S-TFA questionnaire using IBM SPSS Statistics for Windows, Version 28.0 (Armonk, NY: IBM Corp).

##### Data quality

In line with Hobart et al. [19], we evaluated data quality using the proportion of missing data per item and the percentage of computable scale scores; this reflects the participants’ understanding and acceptance of a measure. As recommended by Hobart et al. [19], we considered data quality to be high if the proportion of missing data per item was low and considered a threshold of < 10% as acceptable.

##### Targeting

Following Hobart et al. [19], we evaluated whether the S-TFA questionnaire could target the full variance within the sample and calculated ceiling and floor effects and skewness. Current recommendations suggest that scale floor and ceiling effects should not exceed 15% [19] and that skewness statistics should be within the − 1 to + 1 range [19, 20].

##### Homogeneity

Homogeneity, which refers to scaling assumptions, was assessed using inter-item and item-total correlations. Inter-item correlations between 0.15 and 0.85 [21] and item-total correlation coefficients higher than 0.30 [18] were considered acceptable.

### Ethical consideration

No identifiable/personal data was collected, which was confirmed by the university’s data protection officer. One research team member contacted the participants in the expert panel and collected the CVI questionnaires. These questionnaires were stored following university policy and were handled anonymously within the research team. The participants in the expert and lay panels evaluated the S-TFA questionnaire and the interventions, respectively, from their professional viewpoints as researchers and healthcare professionals. The pilot evaluation of the S-TFA questionnaire was answered anonymously, and no personal or sensitive background information was collected. Further, the lay panels’ interviews were not recorded. Hence, according to the Swedish Ethical Review Act (2003:460), ethical approval was not needed for this study. This was corroborated by the Swedish Ethical Review Authority and the Head for Research and Research Education at the institution where this study was conducted. Nevertheless, all participants were provided verbal or written information about the purpose and procedure of the study; they were also informed that participation was voluntary. All the participants gave verbal or written consent before participating in the interviews or filling in the questionnaire.

## Results

### Face and content validity

Five researchers participated in the expert panel. They all hold a PhD, and three are professors. Eleven healthcare professionals from regional or municipal healthcare participated in the lay panels: two registered nurses, four specialist nurses, a registered nurse with a PhD, two nurse aids, a dietician with a PhD, and a physiotherapist.

Both the expert and lay panels deemed the questionnaire satisfactory in terms of face validity, finding it visually clear and of appropriate length. Further, the lay panels deemed the items’ response options logical and easy to use. However, the translators, experts, and lay panels noted a lack of consistency in response options and an alternation between questions and statements across items. For content validity, the I-CVI ranged 0.2–1, and the following items were above threshold values: Item 2 (How comfortable did you feel?), Item 4 (Moral or ethical consequences), and Item 8 (How confident did you feel?) (see Table 2 for I-CVI). The S-CVI/UA was 0.70 and the S-CVI/Ave 0.60, which are below threshold values. The word comfortable (*illa till mods*) in Item 2 (How comfortable did you feel? ) and the word acceptable (*acceptabel*) in Item 10 (How acceptable was the [intervention] to you?) were both commented on in the free-text by two expert panel members as an possible inadequate translation. Mean I-CVI per rater raged from 2.3 to 3.6.

**Table 2:** Synthesis of protocols from cognitive interviews with healthcare professionals (*n*=11) and evaluations of Item CVI by experts (*n*=5)

Item	Participant reread question or seriously stumbled*	Difficulty generating an answer*	Questioned content of item	Answered a different question from the one that was asked or gave reasoning inconsistent with the answer given	I-CVI
1 Did you like or dislike …	-	-	-	-	0.20**
2 How comfortable did you feel with …	2	3	-	-	1.00
3 How much effort did it take to…	1	1	-	-	0.80**
4 There are moral or ethical consequences with…	1	1	2	-	1.00
5 How fair is… for …	3	1	2	-	0.20**
6 The… has improve …	1	-	-	-	0.80**
7 It is clear to me how … will help to …	1	-	-	-	0.80**
8 How confident did you feel about …	1	1	-	-	1.00
9 … interfered with my other priorities	1	-	-	-	0.60**
10 How acceptable was … to you?	1	4	-	-	0.80**

*Numbers indicate the numbers of healthcare professionals reporting uncertainty and items

**I-CVI below threshold values

### Comprehensibility

In the first round of cognitive interviews, three participants criticized the Swedish wording for comfortable (*illa till mods*) in Item 2 for being old-fashioned language, although no better alternative was given. Further, all participants stopped and reflected on the meaning of fair (*rättvis*) in Item 5, and four of them had difficulties understanding its meaning in relation to the intervention. Further, there were uncertainties concerning whether possible ethical or moral consequences in Item 4 concerned the patients, their personal value system, or the organization. Moreover, four participants found it difficult to answer acceptability (*acceptabel*) in Item 10. We extensively discussed this matter, consulting relevant literature and the TFA’s definition of acceptability and engaging with the expert and lay panel members. However, no superior alternative was identified; therefore, we decided not to revise this item before the psychometric evaluation. In the second round, when the use of different temporal perspectives was evaluated, two participants expressed having difficulty in using the post-intervention assessment, particularly because the intervention took place over a long period of time: ‘It has been a process, and how I feel about the intervention has changed over time.’ Table 2 shows a synthesis of the protocols on uncertainties per item and I-CVI.

### Descriptive pilot evaluation

The healthcare professionals (*n* = 16) reported a median S-TFA score of 30.5 and a mean score of 30.4, with values ranging from 11 to 37 (see Table 3).

**Table 3:** Item-score distribution and item-total correlations for S-TFA questionnaire answered by healthcare professionals (*n* = 16)

Item-score distribution								
Items	Missing data	Mdn (q1-q3)	Floor effect %	Ceiling effect %	Skewness	Kurtosis	*P*-value	ITC
1 Did you like or dislike …	0	5 (3–4)	6	62	-2,73	8,72	< 0.001	0.92
2 How comfortable did you feel with …	0	5 (4–4)	6	81	-3,36	11,95	< 0.001	0.92
3 How much effort did it take to…	0	4 (3–3)	0	19	-1,05	1,18	0.036	0.54
4 There are moral or ethical consequences with…	0	2,5 (2–4)	38	6	0,49	-0,47	0.687	-0.11
5 How fair is… for …	0	5 (2,25 − 4)	0	56	-1,09	-0,17	0.018	0.60
6 The… has improve …	1	4 (3–4)	0	44	-0,55	-0,39	< 0.001	0.76
7 It is clear to me how … will help to …	0	4,5 (3–4)	0	50	-0,65	-0,32	0.002	0.73
8 How confident did you feel about …	0	4 (2,25 − 3)	0	6	-0,19	0.56	0.036	0.55
9 … interfered with my other priorities	0	4 (3–4)	6	38	-1,49	2.12	0.002	0.73
10 How acceptable was the intervention to you?	0	5 (3–4)	6	69	-2,91	9.51	< 0.001	0.94

#### Data quality

The proportion of missing data was low; only one item had missing data from one participant (6%), which is below the acceptable threshold of 10% (see Table 3).

#### Targeting

All S-TFA questionnaire items demonstrated skewed distributions and ceiling effects, except for Item 3 (burden) and Item 8 (self-efficacy), which did not exceed the recommended 15% (see Table 3).

**Fig. 2:**
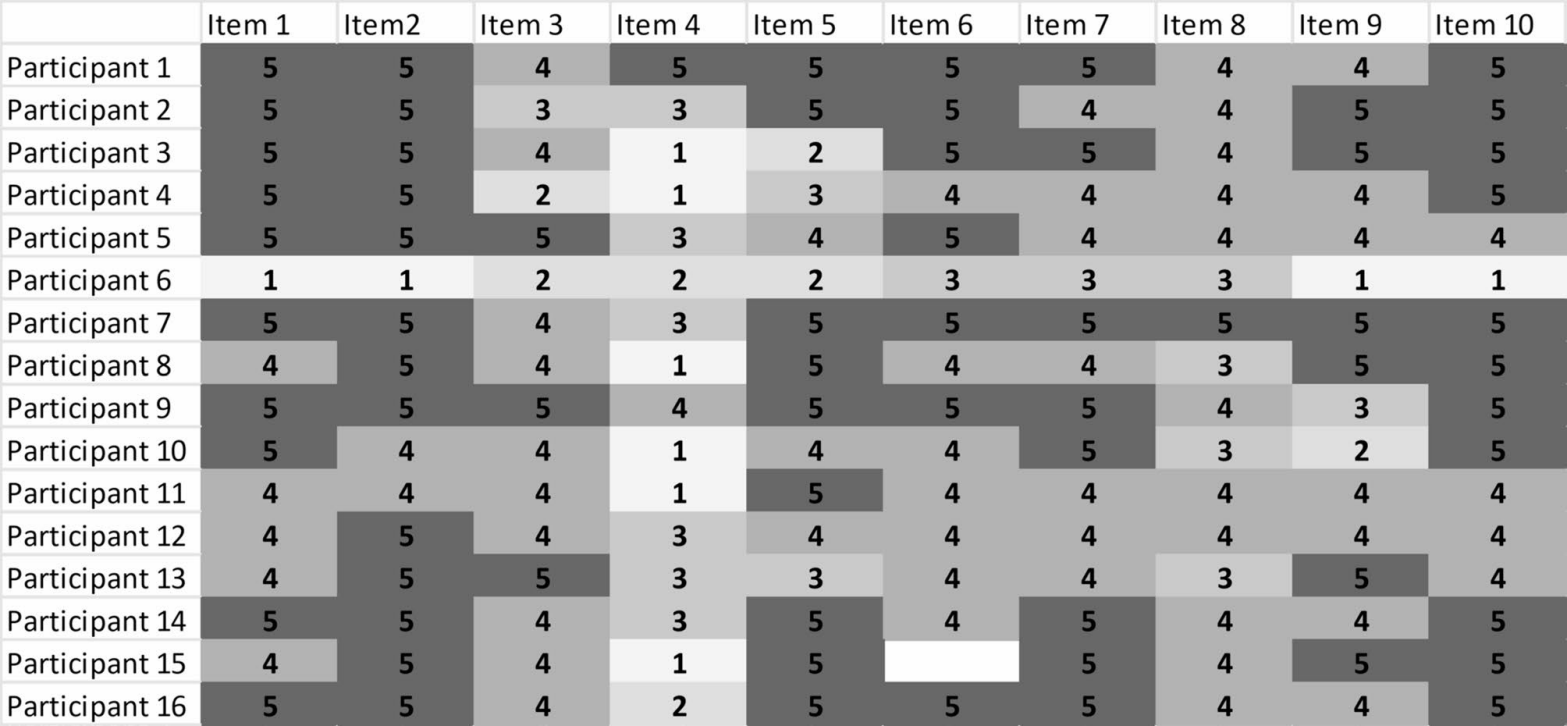
The item distribution for each participant

The results showed that most healthcare professionals scored 3 or 4 for all items except Item 4 (moral or ethical consequences) (see Fig. 2). The distribution of the scale scores was negatively skewed for all items (skewness = − 3.12, see Table 3).

#### Homogeneity

Inter-item correlations were weak for healthcare professionals (mean rho = 0.38, range = − 0.42 to 0.94). The item-total correlation coefficient was below 0.30 for Item 4 (see Table 3), indicating that this item may not contribute effectively to the overall scale.

## Discussion

This study aimed to translate the TFA questionnaire from English to Swedish, culturally adapt it, and conduct a descriptive pilot evaluation of its psychometric properties. Given the importance of acceptability for successful healthcare interventions [1], a rigorously developed, valid, and reliable questionnaire is needed. The demand for such a questionnaire is evident by the extensive use of the TFA questionnaire since its development. However, surprisingly, the existing literature on the application of the questionnaire in non-English speaking contexts is sparse [6]. Studies have emphasized the importance of a thorough translation processes for the validity and reliability of a measure [13, 18, 22, 23]. The opposite risks a shift of meaning, which arguably results in a lack of clarity on what is measured and, consequently, impedes comparisons and synthesis of findings. Therefore, aiming for a valid and reliable Swedish version, we undertook a translation, cultural adaptation, and piloted the questionnaire according to international guidelines [12, 13]. Overall, the results from the evaluations of face validity and comprehensibility support a successful translation and usability. On the other hand, the content validity and comprehensibility evaluations and the descriptive pilot evaluation indicate unresolved issues related to the meaning of acceptability and the structure of the questionnaire. These will be discussed below.

First, the I-CVI of seven items, the S-CVI/UA, and the S-CVI/Ave were below threshold values. This indicates that the items do not reflect the construct being measured: acceptability. Noticeably, there was an inter-rater variation (2.3–3.6): one participant assessed no items as highly relevant and only three items as relevant (Item 2, Item 4, and Item 8), whereas another assessed all items as relevant. This corroborates the previously reported lack of consensus among researchers on the meaning of acceptability [1, 2], emphasizing the need to apply a common framework, such as the TFA, when evaluating interventions. Furthermore, in response to the remarked shifting formulation of items, future studies may explore the possibility of reformulating items (i.e. to either questions or statements) and applying the same response options across items.

Further, the cognitive interviews, in which we elaborated on the application of S-TFA on a variety of interventions, showed that not all items may be suitable for all interventions and settings. This confirms the TFA developers’ approach to the questionnaire: they recommend the consideration of all seven TFA items and the general acceptability item, although in some contexts, not all items will be applicable [7]. Hence, our findings support the developers’ recommendation to researchers to use their judgment and to consult with stakeholder advisors. Since the questionnaire builds on the conceptualization of acceptability as a multidimensional construct with one item per dimension [2, 7], researchers can select the relevant items for the specific intervention being evaluated, as has been done previously [11, 24]. According to the developers of the TFA questionnaire [7], the general acceptability item can be used to explore which of the seven TFA constructs influences participants’ general acceptability judgment. However, the questionnaire’s structure (with one item per dimension) limits the possible conclusions that can be made. Single-item measures can be convenient for research, but they have significant weaknesses in terms of psychometric properties, including lower reliability, limited construct validity, sensitivity to context, ambiguity in interpretation, and potential biases. Thus, single-item measures should be carefully considered, especially when nuanced understanding is essential [25]. We suggest that free-text answers or complementing interviews may strengthen the evaluation of acceptability when using this questionnaire, as was done by Paynter et al. [26].

A relevant finding from the cognitive interviews is that the questionnaire’s structure requires the careful wording of items before use. According to Sekhon et al. [7], researchers need to modify the items regarding tempus, intervention, and engagement. Also, Haydon et al. [5] and Rivera et al. [6] emphasized the critical role of precise wording in their studies. In our study, we did this together with the lay panel in the first round of cognitive interviews, whereas in the following round, we made the modifications before the interview. Based on our experiences from this process, corroborated by literature stressing that established validity and reliability are not transferable to other contexts and populations [12, 18], we support the developers’ recommendation to undertake cognitive interviews with the target population and continue psychometric evaluations when the questionnaire is used in new settings [7], as was done by, for example, Timm et al. [11].

During the cognitive interviews, the relevance of Item 4 was raised by two participants questioned the relevance of Item 4, one participant struggled to formulate a response, and another required a rereading of the question. These challenges may stem from the limited number of interventions the participants have experienced or anticipated that involve moral and ethical consequences in healthcare. Consequently, this item may not be universally applicable across all interventions. Conversely, there are interventions, such as the administration of COVID-19 vaccines, where moral and ethical considerations are highly pertinent [7]. Possible inadequacy of certain items in certain contexts was also reported by Rivera et al. [6]. Therefore, Rivera et al. [6] recommend careful adaptation of sections of the questionnaire that relate to the intervention under evaluation. In addition, there were ceiling effects for most items, which may pose challenges in detecting improvements. This issue requires further investigation and greater awareness among users.

### Strengths and limitations

The comprehensive, systematic, and iterative translation and cultural adaptation process according to international guidelines could be considered a strength in this study. Moreover, we believe the involvement of an expert panel of researchers well-versed in developing, evaluating, and implementing complex healthcare interventions as well as the involvement of different healthcare professionals, both intervention deliverers and receivers from both regional and municipal healthcare, enhanced the internal and external validity of the translation and cultural adaptation process. However, the lack of evaluation among non-healthcare professionals is an obvious limitation of this study. In addition, this study is based on a questionnaire that has not been validated in a large sample, which limits the conclusions that can be made.

Further, this study was based on classical test theory, known to be sample-dependent [18]; thus, psychometric properties could vary between samples. The small sample size (*N* = 16) only allowed descriptive analysis of the questionnaire. As such, further psychometric analysis in larger samples is needed to assess the factors, validity and reliability of the questionnaire. In particular since recent evaluations of the generic questionnaire [6] and the modified versions by Timm et al. [11] and Haydon et al. [5] have revealed different factor structures, pointing to the importance of continued evaluations in new contexts.

## Conclusion

We translated the TFA questionnaire from English to Swedish, culturally adapted it, and evaluated its face and content validity and comprehensibility among experts and lay panels of researchers and healthcare professionals, both intervention deliverers and receivers, across several healthcare interventions. Further, we piloted the Swedish version on a sample of 16 healthcare professionals who had received an educational intervention to find descriptive results that showed scores were negatively skewed. The translation process revealed unresolved issues with content validity, possibly explained by the previously reported lack of consensus on the meaning of acceptability. Nevertheless, the cognitive interviews indicated successful translation.

Overall, the TFA questionnaire seems like a useful brief screening tool for the acceptability of healthcare interventions. Complementing free-text answers or interviews could enhance the understanding of the different aspects the questionnaire addresses. Nevertheless, the generic nature of the questionnaire poses excessive demands on researchers to ensure the questionnaire remains valid and reliable across settings, interventions, and temporal perspectives. We recommend using cognitive interviews and psychometric evaluations in any new setting. In addition, we recommend cross-measure validation between the existing acceptability questionnaires to help further refining the measurement of acceptability.

## Data Availability

The datasets used during the current study are available from the corresponding author on reasonable request.

## Abbreviations

CVI: Content Validity Index
HCP: Healthcare professionals
I-CVI: Item Content Validity Index
S-CVI-Ave: Scale Content Validity Index Average
S-CVI-UA: Scale Content Validity Index Universal Agreement
S-TFA: Swedish Theoretical Framework of Acceptability
TFA: Theoretical Framework of Acceptability

## References

[1] Skivington K, Matthews L, Simpson SA, Craig P, Baird J, Blazeby JM, et al. A new framework for developing and evaluating complex interventions: update of medical research Council guidance. BMJ. 2021;374:n2061. 34593508 10.1136/bmj.n2061 PMC8482308

[2] Sekhon M, Cartwright M, Francis JJ. Acceptability of healthcare interventions: an overview of reviews and development of a theoretical framework. BMC Health Serv Res. 2017;17(1):88. 28126032 10.1186/s12913-017-2031-8 PMC5267473

[3] Williamson P, Altman D, Blazeby J, Clarke M, Gargon E. Driving up the quality and relevance of research through the use of agreed core outcomes. J Health Serv Res Policy. 2012;17(1):1–2. 22294719 10.1258/jhsrp.2011.011131

[4] Dwan K, Altman DG, Arnaiz JA, Bloom J, Chan AW, Cronin E, Decullier E, Easterbrook PJ, Von Elm E, Gamble C, Ghersi D, Ioannidis JP, Simes J, Williamson PR. Systematic review of the empirical evidence of study publication bias and outcome reporting bias. PLoS One. 2008;3(8):e3081. 10.1371/journal.pone.0003081. 10.1371/journal.pone.0003081 PMC2518111 18769481

[5] Haydon HM, Major T, Kelly JT, Catapan SC, Caffery LJ, Smith AC, et al. Development and validation of the digital health acceptability questionnaire. J Telemed Telecare. 2023;29(10suppl):s8–15. 10.1177/1357633X231202279 38007698

[6] Rivera S, Silva-Letelier C, Retamal-Walter F, Fuentes-López E, Contreras J, Marcotti A. Cross-Cultural adaptation and validation of a generic acceptability questionnaire to Spanish. Revista De Investigación E Innovación En Ciencias De La Salud. 2024;7(1):1–19.

[7] Sekhon M, Cartwright M, Francis JJ. Development of a theory-informed questionnaire to assess the acceptability of healthcare interventions. BMC Health Serv Res. 2022;22(1):279. 35232455 10.1186/s12913-022-07577-3 PMC8887649

[8] Sandell T, Schütze H, Miller A, Ivers R. Patients’ acceptance of a shared cancer follow-up model of care between general practitioners and radiation oncologists: A population-based survey using the theoretical framework of acceptability. BMC Prim Care. 2023;24(1):86. 36973691 10.1186/s12875-023-02032-6 PMC10044765

[9] Laing L, Salema NE, Jeffries M, Shamsuddin A, Sheikh A, Chuter A, et al. Understanding factors that could influence patient acceptability of the use of the PINCER intervention in primary care: A qualitative exploration using the theoretical framework of acceptability. PLoS ONE. 2022;17(10):e0275633. 36240174 10.1371/journal.pone.0275633 PMC9565699

[10] Nyumwa P, Bula AK, Nyondo-Mipando AL. Perceptions on acceptability of the 2016 WHO ANC model among the pregnant women in Phalombe district, Malawi - a qualitative study using theoretical framework of acceptability. BMC Pregnancy Childbirth. 2023;23(1):166. 36906538 10.1186/s12884-023-05497-6 PMC10007797

[11] Timm L, Annerstedt KS, Ahlgren J, Absetz P, Alvesson HM, Forsberg BC, et al. Application of the theoretical framework of acceptability to assess a telephone-facilitated health coaching intervention for the prevention and management of type 2 diabetes. PLoS ONE. 2022;17(10):e0275576. 36201441 10.1371/journal.pone.0275576 PMC9536591

[12] Mokkink LB, de Vet HCW, Prinsen CAC, Patrick DL, Alonso J, Bouter LM, et al. COSMIN risk of Bias checklist for systematic reviews of Patient-Reported outcome measures. Qual Life Res. 2018;27(5):1171–9. 29260445 10.1007/s11136-017-1765-4 PMC5891552

[13] Beaton DE, Bombardier C, Guillemin FaBF M. <Guidelines for the process of cross-cultural adaptation of self-report measures.pdf>. Spine. 2000;25:3186–91. 11124735 10.1097/00007632-200012150-00014

[14] Lynn MR. Determination and quantification of content validity. Nurs Res. 1986;35(6):382–6. 3640358

[15] Willis GB. Cognitive Interviewing: a tool for improving questionnaire design. Sage publications; 2004.

[16] Polit DF, Beck CT. The content validity index: are you sure you know what’s being reported? Critique and recommendations. Res Nurs Health. 2006;29(5):489–97. 16977646 10.1002/nur.20147

[17] French DP, Cooke R, McLean N, Williams M, Sutton S. What do people think about when they answer theory of planned behaviour questionnaires? A ‘think aloud’ study. J Health Psychol. 2007;12(4):672–87. 17584818 10.1177/1359105307078174

[18] Streiner DL, Norman GR. Health measurement scales: a practical guide to their development and use. Oxford: Oxford Academic; 2014.

[19] Hobart JC, Riazi A, Lamping DL, Fitzpatrick R, Thompson AJ. Improving the evaluation of therapeutic interventions in multiple sclerosis: development of a patient-based measure of outcome. Health Technol Assess. 2004;8(9):iii, 1–48. 14982653 10.3310/hta8090

[20] Ware JE Jr., Gandek B. Methods for testing data quality, scaling assumptions, and reliability: the IQOLA project approach. International quality of life assessment. J Clin Epidemiol. 1998;51(11):945–52. 9817111 10.1016/s0895-4356(98)00085-7

[21] Clark LA, Watson D. Constructing validity: basic issues in objective scale development. Psychol Assess. 1995;7:309–19.

[22] Sharma A, Pachori H, Das B, Unni D. Methodological rigour in translating instruments - an overlooked yet essential aspect in cross-cultural research. Asian J Psychiatr. 2020;52:102115. 32334397 10.1016/j.ajp.2020.102115

[23] Cruchinho P, López-Franco MD, Capelas ML, Almeida S, Bennett PM, Miranda da Silva M, et al. Translation, Cross-Cultural adaptation, and validation of measurement instruments: A practical guideline for novice researchers. J Multidiscip Healthc. 2024;17:2701–28. 38840704 10.2147/JMDH.S419714 PMC11151507

[24] Kelley-Jones C, Scott SE, Waller J. Acceptability of de-intensified screening for women at low risk of breast cancer: a randomised online experimental survey. BMC Cancer. 2024;24(1):1111. 39243000 10.1186/s12885-024-12847-w PMC11378402

[25] Sarstedt M, Diamantopoulos A, Salzberger T. Should we use single items? Better not. J Bus Res. 2016;69(8):3199–203.

[26] Paynter C, McDonald C, Story D, Francis JJ. Application of the theoretical framework of acceptability in a surgical setting: theoretical and methodological insights. Br J Health Psychol. 2023;28(4):1153–68. 37353989 10.1111/bjhp.12677

